# The Vivarium: Maximizing Learning with Living Invertebrates—An Out-of-School Intervention Is more Effective than an Equivalent Lesson at School

**DOI:** 10.3390/insects9010003

**Published:** 2018-01-02

**Authors:** Peter Wüst-Ackermann, Christian Vollmer, Christoph Randler, Heike Itzek-Greulich

**Affiliations:** 1 Department of Biology, University of Education Heidelberg, Im Neuenheimer Feld 561-2, D-69120 Heidelberg, Germany; wuest@ph-heidelberg.de; 2 Independent Researcher, D-69117 Heidelberg, Germany; vollmer.christian@gmail.com (C.V.); heikeitzek@gmail.com (H.I.-G.); 3 Department of Biology, University of Tübingen, Auf der Morgenstelle 24, D-70726 Tübingen, Germany

**Keywords:** invertebrate education, living animals, achievement, motivation, classroom teaching, school students, pre-service teachers, out-of-school learning

## Abstract

The introduction of living invertebrates into the classroom was investigated. First, possible anchor points for a lesson with living invertebrates are explored by referring to the curriculum of primary/secondary schools and to out-of-school learning. The effectiveness of living animals for increasing interest, motivation, and achievement in recent research is discussed. Next, the Vivarium, an out-of-school learning facility with living invertebrates, is described. The effects of an intervention study with living invertebrates on achievement are then investigated at school (School condition) and out of school (University condition); a third group served as a control condition. The sample consisted of 1861 students (an age range of 10–12 years). Invertebrate-inspired achievement was measured as pre-, post-, and follow-up-tests. Measures of trait and state motivation were applied. The nested data structure was treated with three-level analyses. While achievement generally increased in the treatment groups as compared to the control group, there were significant differences by treatment. The University condition was more effective than the School condition. Achievement was positively related to conscientiousness/interest and negatively to tension. The study concludes that out-of-school learning offers achievement gains when compared to the same treatment implemented at school. The outlook focuses on further research questions that could be implemented with the Vivarium.

## 1. Introduction

### 1.1. Why Living Animals?

There is a concerning decline in interest in school science topics, including animals [1]. Involvement in more experimental tasks could reverse this decline [2], and contact with living animals be a viable way to increase interest in animals in elementary and middle school, even more so because contact with living animals leads to more attention and emotional engagement than museum exhibits [3]. The primary (first-hand) experience with the living animal has many pedagogical advantages and benefits for the students [4]. While the charismatic megafauna is preferred by most students, it is important to also draw attention towards disliked animals to improve environmental concern and to understand their role in nature [5,6]. The direct contact with invertebrate specimens, especially when this direct contact eventually turns out to be enjoyable, can induce an attitudinal and conceptual change from naïve to scientific frameworks [7], resolve misconceptions, and thus reduce cognitive dissonance and make the student more responsive for motivational and achievement gains [8]. Especially working with live invertebrates has emotional aspects and therefore is apt to create a more positive relationship between the students and representatives of this animal class.

### 1.2. The Use of Living Animals Is Recommended by the Curriculum

*Living animals.* Contact with living animals is encouraged in many documents concerned with teaching and learning in secondary schools. For example, the National Association of Biology Teacher (NABT) values the presence of live animals in the classroom with appropriate consideration to the age and maturity level of the students (elementary, middle school, high school, or college): “Live demonstrations and experiments involving animals in precollege education are valuable ways to excite children about science” [9]. In particular, living animals are a firmly anchored topic in the German curriculum on the various school levels, taking into account different focuses. For example, in the German standards for teaching biology [10], the KMK standards are mandatory for all German schools. However, as Germany has sixteen federal states with different types of schools, this paper focuses on the curriculum of the federal state of Baden-Württemberg, Southwest Germany. The educational program described in this paper was developed to suit students in Baden-Württemberg. Here, according to the most recent curriculum, teaching and learning is identical in all Grade 5 and 6 classrooms. These students can range from 10 to 12 years of age. Prior to 2016, students have been separated after Grade 4 into three different stratification levels (lower, intermediate, and upper track (Hauptschule/Werkrealschule, Realschule, and Gymnasium)), usually according to their cognitive abilities. Therefore, during this time, 5th and 6th graders receive one of the three educational levels. Nowadays, they are separated into two different levels, but learning content is rather similar in both stratifications in Grades 5 and 6. Therefore, the educational program developed in this study is suitable for all 5 and 6 graders.), a special emphasis is given to arthropod diversity (p. 43) where students should learn to discover the diversity of arthropods and to make use of taxonomic knowledge.

*Invertebrates.* According to the curriculum of the Ministry of Education Baden-Württemberg [11] (Chapter 3.1.7), teaching invertebrates in Grades 5 and 6 is mandatory with an emphasis on the Western honey bee (Hymenoptera: *Apis mellifera*), the maybug beetle (Coleoptera: *Melolontha melolontha*), and the wood ant (Hymenoptera: *Formica polyctena*). In doing so, the common features and differences within this animal class as well as developmental stages are to be targeted. In addition, a comparison with vertebrate animals is to be drawn and the differences are to be described. Apart from using books, worksheets, and models, the teachers are asked to illustrate these topics with living animals and, to this end, to visit out-of-school learning facilities with their students.

*Out-of-school learning.* The Ministry further notes: “Out-of-school experiences and out-of-school assignments contribute greatly to learning motivation and must therefore be systematically included and highly valued in the evaluation.“ Additionally, they request the maxim “going out of school—bringing something back to school” to increase the effectiveness of school learning [12] (authors’ translation). The Ministry further states: “The immediate nature experience, the observation of nature, the independent practical work, and the students’ problem solving skills shall be the main focus of a good lesson. Primary experience is preferable to secondary experience. For this reason, it is necessary, especially with regard to biological topics, to visit out-of-school learning places” [11] (authors’ translation).

### 1.3. Research Says: Living Animals Are Effective 

Corresponding to this educational policy, live animals and out-of-school settings are highly valued in textbooks on biological education and in scientific research, also and especially in a German context. For example, Staeck [13] recommends the use of living animals in the classroom with a special emphasis on behavioral biology.

*Achievement.* Younger children have lower knowledge about invertebrates—for example, invertebrates (e.g., cockroach, earthworm, snail, stick insect, and centipede) are likely misclassified as reptiles [14]. Therefore, a conceptual understanding of invertebrates, identifying its core concepts and distinguishing invertebrates from vertebrates by studying specific species, should be targeted in a science classroom [15]. Some studies reported that living animals lead to a higher learning outcomes [16,17,18], but Hummel and Randler [19] showed in their meta-analysis that teaching with living animals is only superior to a control group without any teaching, but not inevitably more effective than an intervention based on videos or models. In fourth and fifth graders, knowledge of the Madagascar hissing cockroach (*Gromphadorhina portentosa*) increased more when students were given the opportunity to first observe living specimen and then read about it than when only the text was provided [20]. While Hummel and Randler [19] and Meyer, Balster, Birkhölzer, and Wilde [21] found inconclusive differences in achievement gains in comparison of living animals and a film group, Klingenberg [22] found higher achievement gains in sixth and seventh graders for the group that observed living invertebrates in the classroom. Thus, hands-on activities with living invertebrates in the classroom can increase subject-specific knowledge and lessons with living invertebrates are more effective than a lesson on the same topic without the living invertebrates [17]. However, there are viable alternatives (e.g., film instead of living animal) and basing the lesson on self-determined work may create an additional boost for achievement gains [16,19].

*Interest and motivation.* Exposure to living animals in the classroom is considered worthwhile to spark interest. Concerning cognitive-emotional variables like interest, Hummel, Glück, Rieke, Weisshaar, and Randler [23] found that students are more interested in zoological topics than in botanical subjects. In another study, Hummel and Randler [19] reported no differences between live animals and video presentations in woodlice (*Porcellia scaber*), and snails (*Helix pomatia*), but in a vertebrate species (*Mus musculus f. domestica*). Similarly, Wilde et al. [16] reported higher motivation in experimental groups where living animals were present. In addition, Killermann, Hiering, and Starosta [24] emphasize that living organisms link cognition and emotions, as well as stimulate different senses. In addition, Berck and Graf [25] cast a special focus on the development of interest in biology, which seems to be fostered by living animals and, in turn, may also reduce disgust and anxiety in children and adolescents. The use of living animals in science education is also conducive to the trait motivation of the students [26]. Concerning trait motivation, conscientiousness has the strongest relation to achievement among the Big Five personality traits, and a meta-analysis found correlations of conscientiousness and achievement to be about *r* = 0.24 [27]. Therefore, conscientiousness should be moderately related to achievement outcomes. However, state motivation is expected to be stronger related to achievement than trait motivation [28]. Concerning state motivation, interested students will act more engaged in practical work and be more motivated to learn, invest increased attention, effort, and affect, and therefore will be more academically successful [29]. It is suggested that living animals in the classroom with group work and hands-on activities spark immersion/flow [30], novelty, and surprise, trigger an attention-getting situation, and thus promote students’ interest [16]. For these reasons, motivational trait and state variables should be targeted when investigating achievement outcomes. Another primary goal of the use of living invertebrate animals in science education is to relieve the students’ anxiety and disgust with respect to these lesser known animals. Randler, Hummel, and Prokop [31] report reduced fear and disgust after working with living invertebrates. Klingenberg [22] plead for including “Primärerfahrungen” (primary experiences) in biological learning environments. He justified this with a strong relation to learning with multiple sensorial and emotional aspects [22]. Similarly, Randler, Ilg, and Kern [32] reported that anxiety is related to learning achievement and that high anxiety results in lower achievement.

*Out-of-school learning.* As one of the most important arguments for the frequent visit of out-of-school learning places, one finds the original encounter, which provides primary experiences [33,34], especially with living invertebrates. Usually, the encounter of living animals is inevitably linked with visits of out-of-school places. Out-of-school learning offers immediate natural experience, biological methods with action-oriented and independent, practical work, and social learning [35,36]. Another aspect of living animals in the classroom is that, aside from factual and conceptual knowledge, students acquire “unintended” knowledge (that is, tacit knowledge not covered by the curriculum) as well as procedural and practical skills by exploring and observing the living animals [37]. Out-of-school learning has been proposed to motivate students to become competent and engaged in science topics, to increase students’ interest in taking on a science career by providing authentic scientific tools and practices, to create awareness of the wildlife in the actual world and to make students better citizens and decision makers regarding socioscientific issues [38,39]. While some studies report out-of-school learning to be significantly more effective than learning in school [40], though the out-of-school group observed living animals in this study while the school group did not, other studies found no significant difference and learning in and out-of-school was both equally effective when compared to a control group [41]. Out-of-school learning has motivational advantages and may provide an equalizing environment for all students with less pressure than in school [42,43]; on the other hand, the novelty of the out-of-school environment may reduce its effectiveness. To reduce novelty and to reap its full benefit, out-of-school learning should be embedded into the curriculum at school in a triad starting with preparation at school followed by experimentation out-of-school and debriefing lessons back at school for continuity and sustainable development of learning [43]. Nevertheless, some of the advantages of out-of-school learning can also be created at school when the lesson includes practical work or laboratory activity [44]; bringing living invertebrates to the classroom is only one example for a viable comparison of in- and out-of-school learning. Out-of-school learning always incurs costs, such as travel expenses, and is time-consuming, and bringing live animals to the school may be a worthwhile alternative. Yet, empirical evidence based on sound studies with live animals is scarce. An example is a study on reptiles and amphibians, which resulted in higher achievement and higher choice for the out-of-school group when compared with the school group [40]. The direct contact to living animals can be established by going on field trips to zoos or science museums to observe the animals in their natural habitat or by bringing the living animal into the classroom at school. The present study presents a learning facility on invertebrates, which is special as it can be implemented in as well as out of school.

### 1.4. The Present Study

The present study implemented a parallel group design with a control group to test the effectiveness of learning with living invertebrates in and out of school. The primary dependent variable was invertebrate-inspired achievement at workstations with living animals, which was tested via a questionnaire immediately after the intervention (post-test) and two weeks later (follow-up-test) as compared to a baseline (pre-test). Variables of secondary interest taken on by the present study are state motivation (interest, competence, choice, and pressure/tension), trait motivation (conscientiousness), the students’ rate of completion of their tasks at the workstations, and gender. The present study compared three conditions: learning in school (School condition), out-of-school learning (University condition), and a control condition. The unique aspect of the present study is that the same intervention on living invertebrates was done in school and out-of-school to see which condition performs better.

The present study investigated the following research questions: How does invertebrate-inspired achievement change over time, from pre-test to post-test to follow-up? Do individuals differ in their inter-individual patterns of change in achievement and can differences in these changes be predicted by the presence/absence of treatment or by differences in the treatment, that is, are there differential effects that are attributable to school, classroom, treatment, and student characteristics (between-person differences in within-person change; [45])? More specifically, concerning the treatment, we suppose that there will be an increase in achievement directly after the treatment (post-test), but is this increase in achievement consistent over time (e.g., in a follow-up-test) or will it decline back to lower levels in the follow-up-test?

**Hypothesis** **1.** Invertebrate-inspired achievement is higher after the treatment than before (1a). It is expected that the achievement scores will be highest immediately after treatment and then decline again (1b).

**Hypothesis** **2.** The students in the two treatment conditions (School and University) learn more than in the control condition (2a). Moreover, we expect the achievement gains to be different between the two treatment conditions (School condition and University condition) and one treatment to be superior to the other (2b). However, since previous research was inconclusive whether the in-school or the out-of-school treatment works better, Hypothesis 2 is non-directional.

**Hypothesis** **3.** In the School condition, some classrooms were taught by student experts from the university and some classrooms were taught by their respective biology teacher. We expect no differences between the teachers and expert students because the educational program was identical and the school students worked in most parts on their own, so it is suggested that the teachers/experts only have a weak influence by observing/coaching the students at the workstations.

**Hypothesis** **4.** It is expected that invertebrate-inspired achievement is influenced by motivational variables. Therefore, we included conscientiousness as an indicator of trait motivation assessed prior to the intervention and interest, competence, choice, and pressure/tension as indicators of state motivation assessed after the intervention.

## 2. Materials and Methods

### 2.1. Sample

The sample size was *N* = 1861 students (age range 10–12 years; 51.0% female) of Grades 5 (*n* = 784) and 6 (*n* = 1077) from the intermediate track (Realschule, *n* = 1048) and upper track (Gymnasium, *n* = 813) of secondary education. Participating schools and classrooms were contacted by convenience sampling in Southwest Germany. That is, the established contacts from the collaboration of the University of Education Heidelberg and schools (contacts from the school practice semester and teacher students’ contacts) were used to attract classrooms for the present study. Moreover, there was a note on the website and classes were invited to apply for participation. The study took place from 2014 to 2016. The students were informed that the participation was voluntary and that the questionnaires were only used for anonymous scientific evaluation at the university and therefore unrelated to their grades. The students were instructed on the conduct of living animals and greatest care was employed to not harm the invertebrates during the intervention. This is an educational study that followed the rules of the University of Education. Ethical approval was therefore not obtained. The Declaration of Helsinki does not require any special information in a questionnaires study based on learning and achievement. As a precondition for participation, the teachers/classes agreed to participate in the pre-test, post-test, and follow-up-test. Another precondition for participation was that the students were not to be given any other lessons on invertebrates prior to or during the intervention.

### 2.2. The Vivarium at the University of Education Heidelberg

*History and underlying idea.* The Vivarium of the University of Education in Heidelberg was founded in 1998 as an insect tract *Tropicana* by Prof. Dr. Jürgen Storrer. Since then, it has been integrated in the education of teacher students as well as school students, offering a possibility for teacher students to include living invertebrates in their practical teacher training. From 2014 onwards, it has been renamed Vivarium as since then it has been keeping, breeding, and lending other invertebrate species in addition to insects. Since the winter term of 2014/15, teacher students have been instructed continuously to conduct the school programs as part of the didactic seminars and courses of Peter Wüst-Ackermann. In the winter and summer terms, 4th grade elementary school classes and 5th and 6th grade school classes from schools in and around Heidelberg were invited to the University of Education in order to participate in the 2.5 h “Stationslernen” (“Stationsarbeit” or learning at workstations). For this, they used a newly designed “scientist’s book” (https://tinyurl.com/ydfmohvd) to keep record of their achievement. The workstations aim at experiencing hands-on contact with living invertebrates for secondary teacher education. Thus, the main aim of the program is to benefit both groups, the teacher students and the school students. Furthermore, all living invertebrates could be borrowed free of charge together with the educational resources required for the workstations. The teachers of the classes mentioned above evoked a snowball effect for this educational program, irrespective of whether the program took place at the university or at school.

*Scientific setting.* According to Sauerborn and Brühne [46], the Vivarium is an out-of-school learning place belonging to the category of “living nature” providing a single occasion of contact: The school students visit the Vivarium usually only once [46]. These authors classify out-of-school learning places into two main groups: nature and culture, they then further specify two parts of culture as essential for out-of-school learning: cultural centers and work places. Within these forms of encounter, three forms can be distinguished according to Sauerborn and Brühne [46]: (1) the punctual encounter, (2) the intense encounter, and (3) the project-oriented encounter. The one-to-one meeting is a one-time or isolated contact with the out-of-school learning place. The peculiarity is that the students are not given any further encounters with the same learning location within their school lessons. As a result, the learning and competence objectives should not be overstated. The unique encounter with the place of learning represents the most common method in out-of-school learning, since the rigid teaching hours and rhythms do not allow for more intensive or multiple forms of encounter. The concept of the out-of-school learning place Vivarium fulfills all the characteristics of out-of-school learning places as defined by Gropengießer et al. [34]:

- the original encounter
- authentic concepts
- immediate natural experience
- biological work
- action-orientated and practical work
- project-specific work
- social learning

*Educational policies for the selection of invertebrate species.* At the Vivarium, non-indigenous invertebrates are used. These non-indigenous animals are very impressive at first sight as they display sounds, colors, and movements that are much more impressive than those of indigenous species of the same families that can be found in Germany. Yet they are not endangered species because all of the invertebrates kept in the Vivarium come from commercial breeders and were not caught in the wild. Therefore, one does not have to take into account the Federal Nature Conservation Act and the Federal Protection Ordinance. However, while using living animals in the classroom, one has to act according to the provisions of the animal welfare legislation with regard to a proper animal husbandry [47,48].

The animals should pose no risk for the students as regards safety, hygiene, and health, so the adherence to the “Guidelines on safety in teaching” of the KMK [49] is ensured. The KMK predefined that the selected animals are non-toxic, do not transmit diseases, are appropriate to the species, and are not subjected to animal experiments and that the experiments cause no suffering. Thus, as regards the CITES regulations these animals are not to be reported as none of them belong to an endangered species. In sum, the observed animals were selected for the following aspects and criteria: the invertebrates

- are readily available to be acquired for use in schools,
- are easily taken care of,
- can be easily observed and investigated/handled by students,
- show several aspects of invertebrate diversity,
- illustrate basic biological phenomena (e.g., holometabolism), and
- possess special ecological adaptions (e.g., mimesis).

*The invertebrates at the* Vivarium. Animals of different classes of invertebrates are represented: snails (class: *Gastropoda*), fawn moth and phantom terrors (class: insects), and centipedes (class: *Diplopoda*):

- *Tenebrio molitor* (mealworm beetle) was selected because one can easily observe the four developmental stages i.e., egg, larva (mealworm), pupa, and adult insect (beetle), which represent the holometabolic metamorphosis. Additionally, main features of insects are easily visible, such as their three body compartments (caput, thorax, and abdomen) and their six legs.
- *Ramulus artemis* (Vietnamese stick bug): In this species, leg movement can be observed easily because of the size of the animal (8–12 cm) and the slow movements of their legs. Furthermore, the Insect’s legs morphology (coxa, trochanter, femur, tibia, and tarsi) can also be viewed very easily. In addition to that, the main features of insect are addressed again (as a repetition). In this insect, the difference to the metamorphosis of the mealworm beetle is stated, observing the hemi-metabolic metamorphosis (without diapause) by examining several larval stages of different sizes.
- *Archispirostreptus gigas* (giant African millipede) and *Spirostreptidae* sp. *6* (Madagascar ringed millipede). In the species mentioned first, the students try to estimate the number of legs, which are of course less than 1000 (usually around 200). However, students can easily figure out why the millipede was given its name. They are also able to observe and describe the wave-like coordination of their many legs while moving forward.
- *Gromphadorhina portentosa* (Madagascar hissing cockroach). Students reason about their adaptation to their environment because of the impressive sound and their quick movement. Additionally, abiotic factors (especially temperature) are one topic that is addressed in this species. For the various uses of the Madagascar hissing cockroach in the classroom, see also the research by Wagler [50,51,52,53,54,55].
- *Lissachatina fulica* (giant African land snail) was chosen because it is a mollusc species and the wave-like movement of its muscular foot can be easily observed through a glass pane. Students can observe their special ability to crawl over sharp objects and look at their rasping tongue (radula). Being one of the largest land living snails of the world with a shell length up to 20 cm and a body length of 30 cm maximum, they display every morphological feature in a way that is easy to observe.
- *Heteropteryx dilatata* (jungle nymph) is perceived as one of the heaviest and most curious insects of the world with its size limited by the tracheae system (restricted by turbulent and laminar oxygen flows). Because of its enormous body size (approximately 15 cm) and weight (approximately 40 g), the tracheal (respiratory) system in general and the mandibles are clearly visible while feeding. Moreover, the morphological features of the insect`s body and legs can be observed at its best. This species also displays sexual dimorphism as the females are much larger than the males, and the wing pairs of the females are shortened. They lay the biggest insect eggs (around 8 mm), which are camouflaged with the ground due to their dark brown coloring. The animals themselves also camouflage with their habitat, resembling green, thorny, and leafy twigs, moving with the plant not being seen and consumed by their enemies, thus displaying an obvious survival strategy of mimesis that can be easily observed.

**Figure 1:**
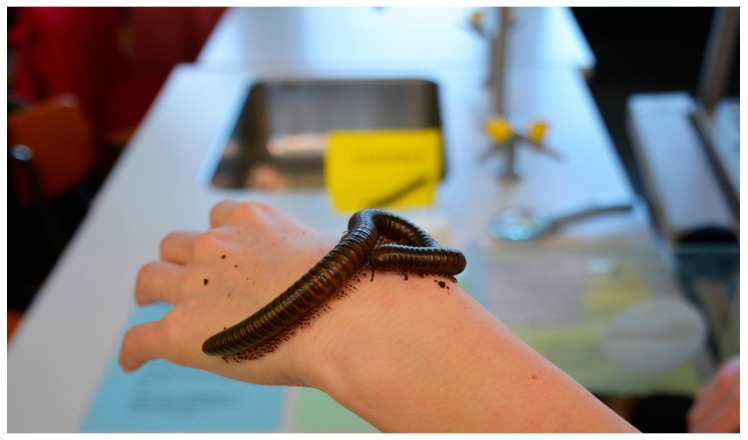
Crawling millipede (*Spirostreptidae* sp. 6).

**Figure 2:**
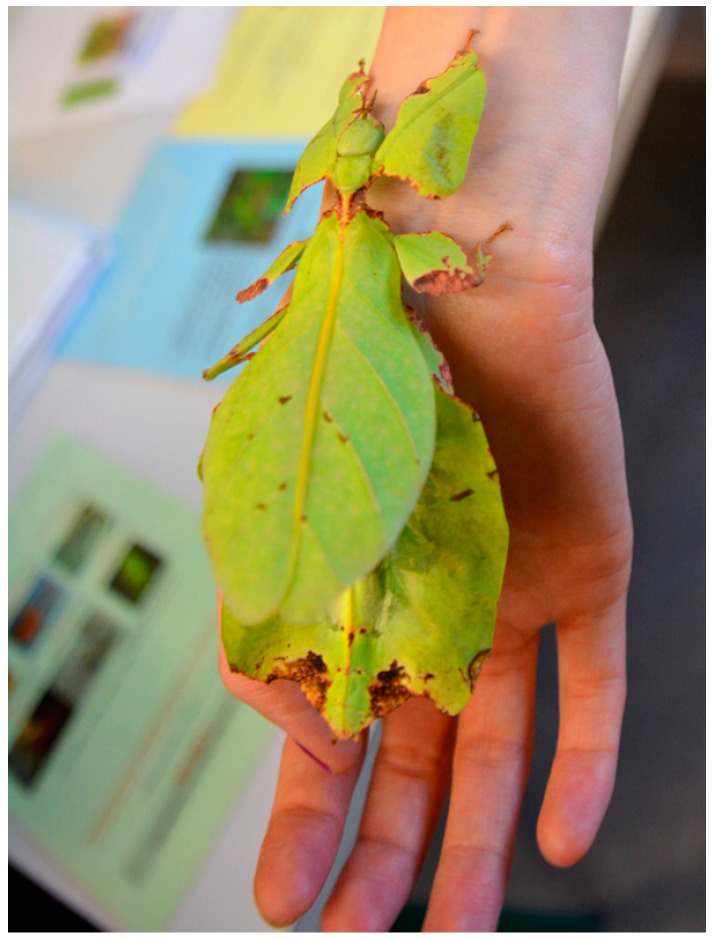
Crawling insect (*Phyllium giganteum*).

**Figure 3:**
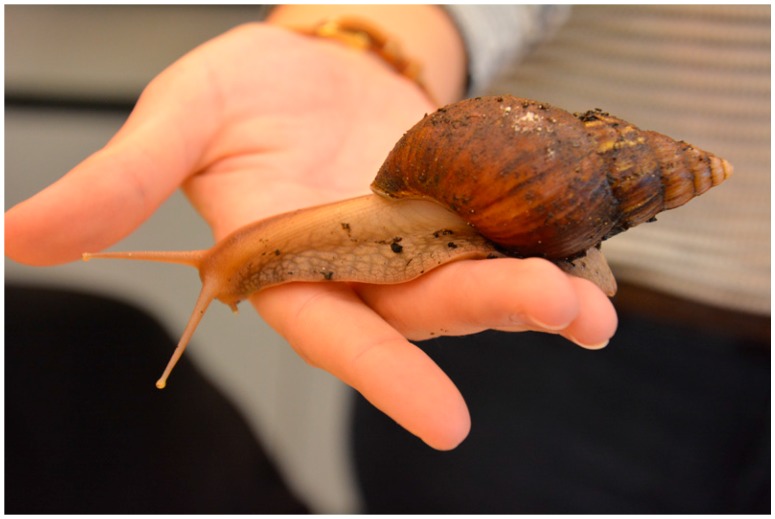
Giant African land snail (*Lissachatina fulica*).

The main aspects for choosing phasmids are based on their behavioral and morphological adaption. As mentioned above, these species camouflage with their habitat, resembling twigs, also showing a back-and-forth movement imitating the wind moving the vegetation and thus displaying mimesis. Other species living at the *Viviarium* include *Eurycantha calcarata* (a thorny devil stick insect), *Phyllium giganteum* (a giant Malaysian leaf insect), and *Extatosoma tiaratum* (a giant prickly stick insect). All these species display sexual dimorphism and mimesis. These animals are easy to keep and breed at the University facilities. They can be easily transported in their terrariums. They are easy to handle and transfer as regards their size and movement pattern (see Figure 1, Figure 2 and Figure 3).

This way, all animals are suitable for an original encounter with the animals on hand; e.g., the student in Figure 1 can feel the wave-like pattern of the millipede movement and can hear its scratchy sound. The student in Figure 2 can feel the leaf insect’s rough legs and experience its sheer size, green color, and leafy shape. The student in Figure 3 can feel the snail’s crawling, moist, and slimy foot moving across his hand. Thus, the direct interaction with the live invertebrates elicits different senses such as visual, acoustic, haptic, and olfactory senses. Experience shows that the initial restraint (anxiety/disgust) decreases when the students interact with the invertebrates and that disgust can even turn into fascination. The direct interaction with the live invertebrates in hand allows perception and attention processes, which may turn the initial anxiety/disgust into calmer or positive emotions. However, the Vivarium has not been used for such studies before. The animals living at the Vivarium receive a high degree of emotional attention and address several senses of the students when they observe and experiment at the stations.

### 2.3. The Vivarium Workstations

**Figure 4:**
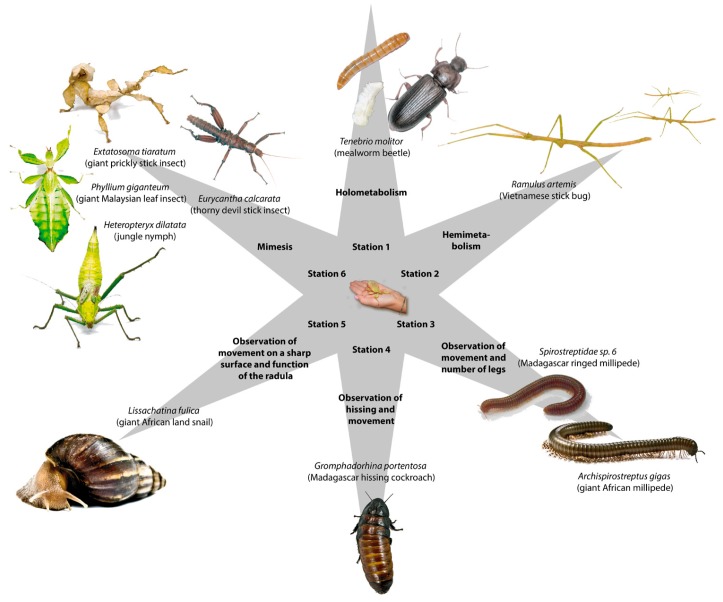
Topics of the workstations at the Vivarium.

“Stationslernen” or workstations are often used in biology classroom settings [56] as well as in out-of-schools settings, such as zoos [57]. Altogether there were six stations for instruction with the invertebrates. Figure 4 gives an overview of each station, with different research focuses. Stations 1 and 2 were concerned with the characteristics and the development of insects. At Station 1, the mealworm beetle (*Tenebrio molitor*) was used to investigate the complete holometabole development of insects. At Station 2, the students were able to observe the Vietnamese stick bug (*Ramulus artemis*), which presented an example of the incomplete (hemimetabole) development of insects. The third station dealt with the African giant millipedes (*Archispirostreptus gigas*) and the question of whether this is an insect or not. At the fourth station, the students were given the opportunity to hold the Madagascar cockroach (*Gromphadorrhina portentosa*) in their hands and could see how fast it can run and listen to the hissing. The fifth station was about the giant land snail (*Lissachatina fulica*). The snail could be observed crossing shards and razor blade edges without hurting itself. Then, the students were allowed to feed the snails with a special mixture of sugar, wheat flour, and water and thus experience the sensation of a rasping radula at their fingertip. This taught them in an impressive way how snails can feed on cucumber slices as well as hard substances such as egg shells. The sixth station dealt with the subject of miming and mimicry (camouflage/protection against predators), which was illustrated by the giant leaf insect (*Phyllium giganteum*), the giant prickly stick insect (*Extatosoma tiaratum*), the devil stick insect (*Eurycantha calcarata*), and the jungle nymph (*Heteropteryx dilatata*). The “Stationslernen” can either be held in the seminar rooms of the University or brought to school and set up in any biology lab there. Any biology teacher is able to carry through the program at school after having been instructed by the author or having visited the Vivarium at the university with one of their classes.

At the beginning of the program, the students are sent into groups of four or five either by the expert students or their teacher, randomly using lots with numbers on paper slips, and each group is seated at their first station according to the number they had drawn. At the beginning of the work at the stations, the students are given a 6-page notebook (“scientist’s book”) to take notes on their observations. Based on our previous work at zoos [40,57,58,59], we consider using a “scientist’s book” as a motivating tool for the students and, in addition, a good possibility in terms of retaining the learning content. While working at their station, the students stay within their group, making their way through all workstations one after the other within a given time period of 15 min for each station, changing their workstations in a clockwise direction each time a signal is given by the teacher/teacher student (expert). Each station displays at least one species of living invertebrates and all supplementary materials that are needed to complete the task, e.g., color-coded leaflets with information, tasks, and solutions as well as tools to interact with the animals and safety devices such as gloves or forceps.

Using the “workstations” method as the basic structure of the program was based on both pedagogical and animal ethical considerations as well as structural reasons. From a pedagogical point, learning in small groups is more effective than in a typical classroom setting [60]. Secondly, separating the classes into six different workstations allows us to place every animal group at a different place. This method is applied during the program rather than group work where each group deals with all materials one after the other staying at their place. Thus, the amount of materials and animals are minimized. An appropriate space (1–2 m) between the workstations is maintained to allow the students to minimize interference from other stations and thus enable them to discuss their findings appropriately. After the students have finished the program, they are allowed to take some pictures of their favorite invertebrates. This is also considered to increase the involvement of the students. However, this has not been tested.

*Acquired competencies and skills.* The work carried out at the workstations promotes scientific competencies and the acquirement of practical work methods and skills through diverse challenges for the students. The areas of competence include knowledge acquisition, “knowledge discovery”, and communication skills. The students are encouraged to observe and compare very closely and to describe their findings. While working with living invertebrates, ethical aspirations and cognitive interest have to be considered. On the one hand, animals should be respected and stressed as little as possible; on the other hand, there is also an interest in attaining knowledge about them.

### 2.4. Study Design

**Figure 5:**
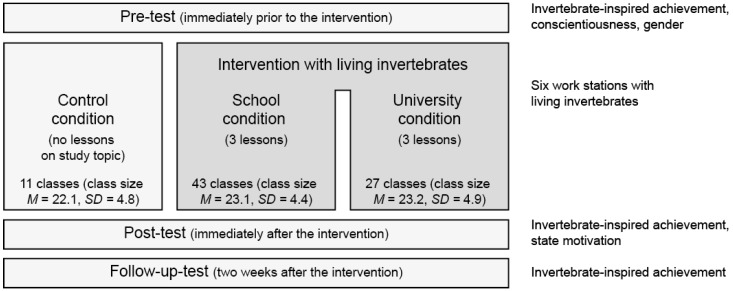
Study design.

The study included a pre-test, the intervention, a post-test, and a follow-up-test. There were three conditions with different treatment: the control condition, the School condition, and the University condition (Figure 5). For all three conditions and prior to the pre-test and post-test questionnaire, photographs of nine invertebrates were displayed in the classroom (http://www.ph-heidelberg.de/tiere.html). The students filled out the questionnaires on pre-test, post-test, and follow-up-test. The control condition did not receive any treatment other than the photographs of the invertebrates shown prior to the pre-test and post-test. The School condition received the intervention at school, while the University condition received an equivalent intervention at the university.

*The treatment (School condition and University condition).* The treatment was always implemented by two teacher students (“experts”) experienced in the handling of the invertebrates with the exception of five classes of the School condition that were not taught by the two experts but by the students’ respective biology teachers. The experts had some training before the intervention started. They took part in a seminar at the University of Education Heidelberg where, prior to the intervention, the experts practiced the treatment together with other teacher students and one of the authors. The invertebrate breeding facility “Vivarium” at the university was designed for this purpose, and students of Grades 4–7 could either visit the university or borrow the living invertebrates for study at the school. An animal care attendant took care of the invertebrates and the transport to the schools.

The intervention was based on guided inquiry and a student-centered perspective of learning (scaffolding; [61]). The two experts (or in the case of the five classes of the School condition, the teachers) acted as observing coaches and were available for answering any questions that occurred during the work at the stations. The students worked independently in groups and to a large degree without interference of the experts or teachers. For the workstations, the students were sorted into groups of three to five to the stations at random using lots (numbers on slips of paper). Prior to the workstations, the students were instructed on the behavior of the living animals and rules of conduct regarding the living animals. There was a given time span (15 min), which was the same for each of the six stations, and a signal was given when it was time to swap the stations. Each station included living invertebrates, tools to interact with the invertebrates, and color-coded leaflets with information, tasks, and solutions. Moreover, at the beginning of the work at the stations, the students were given a notebook (a “scientist’s book” (http://www.ph-heidelberg.de/ausserschulischer-unterricht.html)) to take note of their observations. The fifth and sixth graders were asked to finish all six stations clockwise. At school, the teacher in charge provided a classroom with the technical equipment needed for the intervention (classroom with movable tables). The intervention took place in the school students’ regular biology classroom. The study conduct was audited by one of the authors.

### 2.5. Measures

The questionnaire included an achievement test (9 items, see Table 1; implemented at the pre-test, post-test, and follow-up-test), a conscientiousness scale (2 items; [62]; implemented at the pre-test), and a scale on state motivation (12 items; 3 items on interest, competence, choice, and pressure/tension, respectively; [63]; implemented at the post-test). Moreover, as a measure of implementation fidelity in the two treatment groups, at the post-test and for each station, the students were asked if they did complete the station (yes/no). The students’ gender was recorded as part of the personal code, which was used to link the three questionnaire waves.

**Figure 6:**
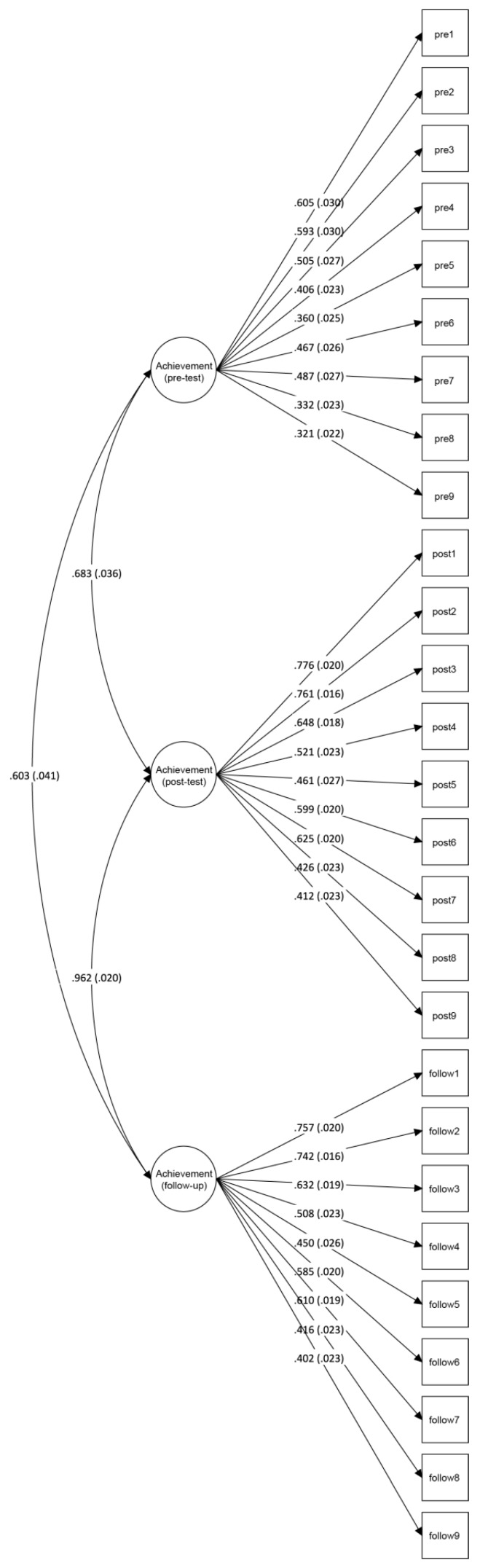
Invertebrate-inspired achievement test, confirmatory factor analysis. *Note: N* = 1861; model fit information: χ^2^/df = 12.3, RMSEA = 0.078 [90% CI = 0.076, 0.080], CFI = 0.690, TLI = 0.677.

For the achievement test, a confirmatory factor analysis for categorical items with WLSMV estimator was performed in Mplus 7 [64]. Factor loadings (standardized regression coefficients with standard errors) and model fit information are shown in Figure 6. The latent factors at post-test and follow-up-test were highly correlated (*r* = 0.962) and the items had higher factor loadings than in the pre-test. The latent factors at pre-test and post-test were moderately correlated (*r* = 0.603) which indicates some temporal consistence between pre- and post-test. However, since this correlation is not high and the factorial structure was better at the post-test and follow-up-test, this suggests that students improved their knowledge from pre-test to post-test. Cronbach’s α, calculated with SPSS 24 [65], was 0.395 for the pre-test, 0.676 for the post-test, and 0.667 for the follow-up-test.

### 2.6. Data Analysis Strategy

Data analyses started only after the data collection was completed and there were no interim analyses. For the preliminary analyses, *t*-tests, analysis of variance with Bonferroni correction, Pearson’s correlations, as well as Figure 2 and Figure 3, were done in SPSS 24. Longitudinal three-level regression analyses with testing time nested in students and classrooms were calculated in Mplus 7 to investigate Hypothesis 1. The data matrix was converted into the person-period format to analyze the effects of testing times (pre-test, post-test, and follow-up-test) on invertebrate-inspired achievement in regression analyses, with testing time and squared testing time as independent variables. Three-level regression with students clustered in classrooms and schools were again conducted in Mplus 7 to investigate Hypotheses 2 and 3 using the person-level data format for the achievement scores at post-test and follow-up-test separately. The continuous variables were z-standardized for the three-level regressions and dummy variables were used for the numerical variables gender, school type, and the treatment conditions (University condition and control condition). The School condition served as the reference group. The explained variance (R^2^) for the three-level models was calculated as 1-(sum of the residual variances of the baseline model/sum of the residual variances of the respective model; [66]). There was attrition on the class level. One class from the University condition did not fill out the pre-test, two classes from the control condition did not fill out the post-test, and two classes from the University condition and three classes from the School condition did not fill out the follow-up-test (see Table 2).

## 3. Results

### 3.1. Preliminary Analyses

**Figure 7:**
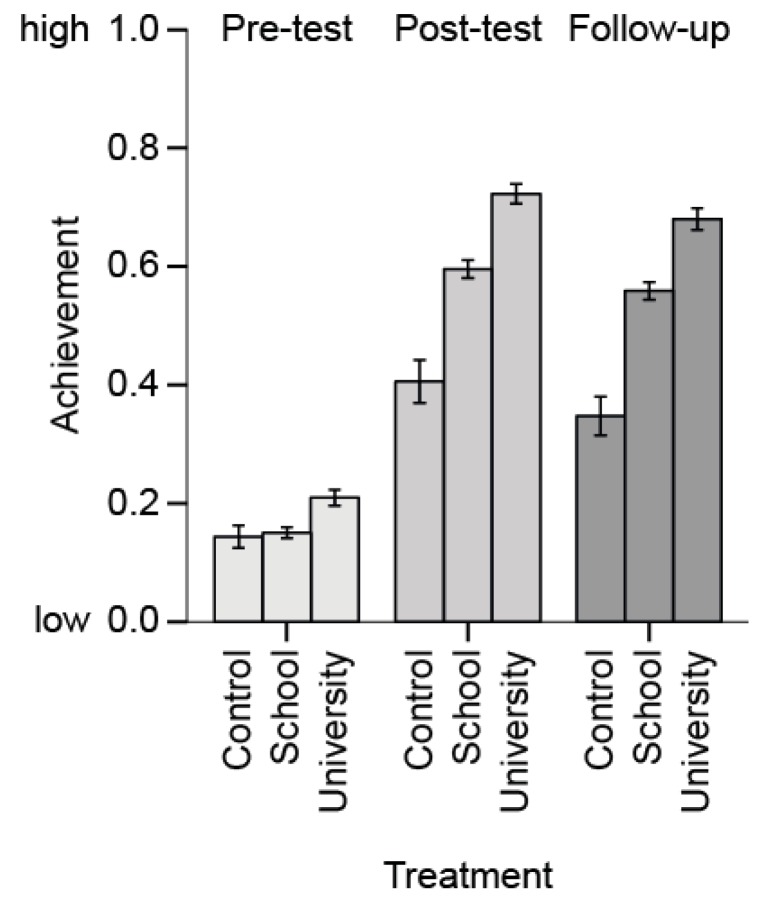
Invertebrate-inspired achievement by treatment and testing time. Note: Error bars 95% CI.

Pre-intervention differences in achievement across the three groups (School condition, University condition, and the control condition) were investigated via ANOVA post-hoc tests with Bonferroni correction and the University condition had significantly higher pre-test achievement scores (*M* = 0.20; *p* < 0.001) than the School condition (*M* = 0.15) and the control condition (*M* = 0.14; see Table 2). While all three conditions increased invertebrate-inspired achievement with higher scores on post-test and follow-up-test, in the post-test and follow-up-test, achievement was higher in the two treatment conditions compared to the control condition (Figure 7). Concerning the attrition on the class level at the follow-up-test, the two classes from the University condition that did not fill out the follow-up-test performed significantly worse in the invertebrate-inspired achievement at the post-test than classes from the University condition that did fill out the follow-up-test (mean difference = 0.14; *p* < 0.001), but there was no significant effect for this difference in the three missing classes from the School condition (mean difference = 0.05; *p* = 0.249). The invertebrate-inspired achievement in the five classes of the School condition that were taught by their respective biology teachers (post-test: *M* = 0.62 [*SD* = 0.23]; follow-up-test: *M* = 0.58 [*SD* = 0.22]) did not significantly differ from the other classes of the School condition taught by the experts from university (post-test: *M* = 0.58 [*SD* = 0.23]; follow-up-test: *M* = 0.55 [*SD* = 0.22]; two-level analysis with class as cluster variable and controlling for pre-test achievement and school type; post-test: *p* = 0.550; follow-up-test: *p* = 0.477).

The completion rate for the workstations was higher in the University condition (88%) than in the School condition (84%; *p* = 0.01). Invertebrate-inspired achievement (post-test and follow-up-test) was positively related to conscientiousness, interest, competence, and choice; on the other hand, it was negatively related to pressure/tension (see the correlation coefficients in Table 3). Moreover, students from the two treatment conditions who completed the work at the stations showed higher achievement and motivational scores than students who did not finish some of the stations (Table 3).

### 3.2. Effects of the Treatment on Invertebrate-Inspired Achievement (Longitudinal Three-Level Regression)

**Figure 8:**
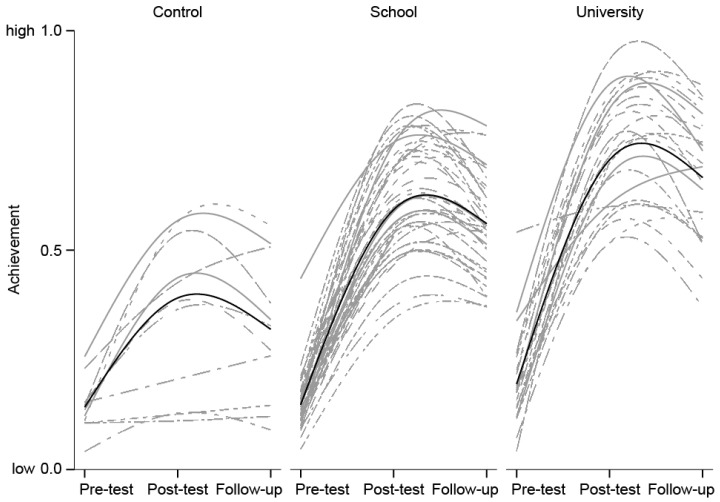
Invertebrate-inspired achievement by treatment and testing time, trajectories on the class level. Note: Spline interpolation; black lines are averages per condition; gray lines are averages per classroom.

While invertebrate-inspired achievement increased from pre-test to post-test, after the peak at the post-test, achievement declined again, but this decline was flatter (see Figure 8); thus, apart from a linear increase in achievement, this also implies a curvilinear (negative quadratic) effect. Both the positive linear and the negative quadratic effect of testing time were highly significant—linear: *R*^2^ = 0.31 (Model 1, Table 4). The negative quadratic effect added another 16% of explained variance to an overall total *R*^2^ of 0.47 (Model 2, Table 4), indicating a decline of invertebrate-inspired achievement from post-test to follow-up-test. A multi-group analysis (Table 4) confirmed that the linear/quadratic effect (Model 2, Table 4) was strongest for the University condition (59% explained variance) and weakest for the control condition (23% explained variance).

### 3.3. Further Predictors of Invertebrate-Inspired Achievement (Three-Level Regression)

The intraclass correlations (*ICC*) of invertebrate-inspired achievement was 0.17 for the class level and 0.23 for the school level at post-test and 0.18 for the class level and 0.23 for the school level at follow-up-test, suggesting that three-level analyses that take the clustered nature of the data (students nested in classrooms and classrooms nested in schools) into account are required. The *ICC*s for the comparison of the two treatment conditions (excluding the control condition; Model 3, Table 5) were 0.15 for the class level and 0.16 for the school level (post-test) and 0.17 for the class level and 0.11 for the school level (follow-up-test). The effects of the intervention and other predictors on invertebrate-inspired achievement were thus investigated via three-level regressions with classroom and school as cluster variables at two time points (post-test and follow-up-test). For both time points, three models were specified.

Model 1 (Table 5) investigated differences in invertebrate-inspired achievement in comparison of the three conditions (the University condition, the School condition, and the control condition). Students in the University condition had significantly higher invertebrate-inspired achievement than the School (*p* = 0.014) and control conditions at the post-test but did not differ from the School condition at the follow-up-test (*p* = 0.056), while the control condition had significantly lower invertebrate-inspired achievement than the School condition (post-test, *p* = 0.003; follow-up-test, *p* < 0.001) and the University condition. This indicates that, while both interventions were effective in comparison with the control condition, students in the University condition learned significantly more than students in the School condition, but this difference was only significant at the post-test.

Model 2 (Table 5) added the following covariates: gender, conscientiousness, interest, competence, choice, pressure/tension (on the student level), and school type (on the school level). Girls learned significantly more than boys. Moreover, conscientiousness had a significantly positive relation to invertebrate-inspired achievement, while pressure/tension was significantly and negatively related to invertebrate-inspired achievement. Interest and choice had a positive effect on the post-test only but not on the follow-up-test, and perceived competence was unrelated to invertebrate-inspired achievement. On the school level, the school type was significantly related to invertebrate-inspired achievement with students from the upper track sharing higher achievement scores than students from the intermediate track.

Model 3 (Table 5) compared the two treatment conditions only (School and University). Moreover, “station completed” was introduced as an additional covariate. The results were quite similar to Model 2 (except for the non-significant “interest” at post-test) and “station completed” had highly significant effects on invertebrate-inspired achievement at post-test and follow-up-test, indicating that students who completed the workstations showed higher achievement scores than students who did not complete some stations.

The effects were generally stronger at the post-test than at the follow-up-test. While Model 1 explained 19% of variance at the post-test and 21% at the follow-up-test, the addition of the covariates (Model 2; Table 5) lead to 32% of variance explained at the post-test and 29% at the follow-up-test, while 31% (post-test) and 20% (follow-up-test) of variance were explained by the comparison of the two treatment conditions in Model 3. 

## 4. Discussion

The effects of an intervention study with living invertebrates on invertebrate-inspired achievement were investigated. The intervention was implemented in two different settings: one condition received the treatment at school and another condition received the treatment in an out-of-school context; a third group received no treatment and served as a control condition. While achievement generally increased after treatment and then declined again, there were significant differences in comparison of the three conditions: both treatment groups had significant gains in achievement when compared to the control group, and the University condition performed better than the School condition.

*Achievement in and out of school.* Invertebrate-inspired achievement was higher at the post-test than at the pre-test, confirming Hypothesis 1a. The trajectories of invertebrate-inspired achievement were composed of a linear increase and a curvilinear effect that indicated a decline in invertebrate-inspired achievement from post-test to follow-up-test, confirming Hypothesis 1b. The treatment was effective in both treatment conditions (School and University) and significantly more effective than in the control condition, confirming Hypothesis 2a. The invertebrate-inspired achievement gains in the two treatment conditions differed significantly (Hypothesis 2b) and the gains were larger in the University condition than in the School condition. The larger achievement gains in the out-of-school condition was a result that contributes to the field of research because of the unique setup of the study: the use of living animals in school was compared with that out of school, which, to our best knowledge, had not been done before, so the results are interesting. It was surprising to see that the out-of-school setup fared better than the in-school setup, even more so because, in another context that compared in-school and out-of-school learning (chemistry of starch; [42]), the achievement was higher for the School group. There were no differences in invertebrate-inspired achievement between the teacher-lead and expert-lead classes in the School condition (Hypothesis 3); this indicates either that there was little interference and that the students managed to do the tasks at the workstations on their own or that the experts and the teachers did not differ in their teaching style. Girls showed higher achievement after treatment than boys, which was expected since previous research found girls being at an advantage in science subjects (effect size *d* = 0.15; meta-analysis [67]).

*Motivation.* There were significant effects of motivational variables on invertebrate-inspired achievement (Hypothesis 4). Conscientiousness was positively related to achievement, which was expected, but the correlation was somewhat lower than suggested by the meta-analysis [27], which can be due to the young age group of the present study. Nevertheless, in the three-level regression (Models 2–3), conscientiousness was a significant predictor of invertebrate-inspired achievement in the post-test and follow-up-test, confirming that this aspect of trait motivation was a significant predictor of achievement while controlling for all other study variables. The correlations with achievement were stronger for state motivation than for trait motivation, which confirms results of earlier research for achievement goals [28] and was expected, because the state measure of motivation occurred immediately after the intervention and was directly connected to the content of the lesson.

### 4.1. Limitations

First, the Vivarium was an ongoing project at the University of Education Heidelberg, and schools were invited to participate over the span of three years. Partly due to this long timeframe, the schools and classrooms were not randomly selected and not randomly attributed (e.g., using computer-generated random numbers) to the three conditions and there was no allocation concealment mechanism or blinding of study participants and scientists implemented. Very similar to the real school context, the teachers decided if they wanted their class to participate and whether they preferred the intervention to be done at the school or at the university. Therefore, the present study is not a randomized controlled field trial. However, the intervention had high ecological validity.

Second, the control condition had considerable gains in achievement although they received no treatment. This can be either attributed to a re-test effect or to some kind of “contamination” of the control group. One cause for achievement gains in the control condition could be that the students of the control group were shown photographs of the invertebrates prior to the questionnaires so that the students at least could associate an animal with the species name of the invertebrates that occurred in the questionnaire. One cause of contamination could be that the teachers from the control condition got interested in the topic and there was some kind of information given by the teacher to debrief the questionnaire, which was not intended to happen and was discouraged by the experts—it was recommended that this information be delayed until after the follow-up-test was done. Another cause of contamination of the control condition could be that the students themselves got interested in the topic and did some research on invertebrates on their own in their spare time. The present study did not control for these possible contaminations of the control group and we recommend future studies to include questions on what happened between the pre-test and post-test to control for any circumstances that could influence the outcomes.

As another limitation, there are many other invertebrate species worthwhile to be included in workstations. For example, we did not implement spiders into our teaching, despite the well-known positive effects of using spiders in the curriculum [68,69]. Additionally, the position and attitude of the teachers may have had an influence because the teachers and schools were a self-selected sample. Thus, teachers disliking invertebrates may not visit the Vivarium nor include these animals in their school teaching. However, this issue cannot be solved in regular school teaching.

Apart from these limitations, other limitations generally found in school intervention studies also apply. The results are not generalizable across different topics and different out-of-school learning environments.

### 4.2. Practical Relevance

The out-of-school condition at the University of Education Heidelberg acquired higher invertebrate-inspired achievement than the School condition. This indicates that out-of-school learning is a worthwhile endeavor and that, when learning with living animals, out-of-school learning contributes an additional unique aspects that fosters learning. These aspects can be related to the different and new surroundings that keep students’ interest in the topic high. Another aspect could be that, at the out-of-school condition, the students act more freely or are more motivated to invest their time in learning because the out-of-school environment is more meaningful to them because it is more related to the “real” world. The results from the present study therefore suggest that classrooms should go on field trips to explore the world to come into contact with nature and to attempt new scientific approaches, because field trips can increase motivational and learning outcomes.

Interdisciplinary teaching projects could also profit from the Vivarium, for example, the live invertebrates could be implemented in teaching in religion class (the biblical plague of insects), in geography class (climate), ethics class (invertebrates as food for humans), sports class (animal flow), engineering class (invertebrates as model for robotics), chemistry class (glyphosate, soil pH-value), mathematics class (snail shell and log rhythmic scale), or language class (creative writing). The Vivarium could also be converted into a mobile truck to improve its versatility in providing educational resources for schools.

Future studies that implement this study design could do the pre-test either in a separate room or during a lesson prior to the intervention to avoid unnecessary student distraction by the trial settings. Moreover, each class could be divided into a half. Each group could then be educated either at university or school to be able to reach a higher level of comparability in our results. However, it would require additional personnel costs to apply the intervention to the smaller groups and the smaller group size could have its own (presumably positive) effect on the invertebrate-inspired achievement outcome. Furthermore, this would in turn diminish some of the ecological validity of the study, because, in the actual world, classes are not usually split.

### 4.3. Outlook

Further experimentation with the invertebrates at the Vivarium may include observation of the mimicking movement of the *Extatosoma tiaratum*, imitating a dry eucalyptus leaf in the wind, or observation of the phasmid’s adhesive organ when placed on a glass plane, hanging upside down or vertically [70] to determine the rate of assimilation of *Extatosoma tiaratum* [71] or to investigate the olfaction and the temperature tolerance of *Gromphadorhina portentosa* [72]. In addition to this, we would like to provide schools with educational resources and live animals for the implementation of long-term observation/experiments on the topics population dynamics and ecology of phasmids. This way, the students could care for the phasmids over a longer time frame (e.g., 1–3 months) and study their population dynamics. The same kind of experimentation could be done with *Tenebrio molitor* specimens, which are easier to keep than the phasmids. Example experiments include studying the population dynamics, studying the population in different environments (light/dark), why *Tenebrio molitor* specimens dig into the ground, and how much *Tenebrio molitor* larvae eat [73]. A further research question for the students with *Tenebrio molitor* would be whether they eat plastic and to discuss the environmental consequences [74].

Moreover, there are some research questions that will be addressed in future studies dealing with the Vivarium and other science center outreach labs. One aspect is the influence of emotions in different settings and the question of whether disgust and anxiety can be reduced by such educational interventions, as, e.g., found by Prokop et al., Prokop and Fančovičová, Randler et al. [17,58,75], and Wagler and Wagler [68]. Further interesting questions include aspects of involvement such as whether the handling and touching of live animals may reduce fear and anxiety and increase involvement in learning. This has been rarely addressed, e.g., in studies with snakes [76]. In this case, one could compare the experience of living animals versus the same teaching but with the addition of touching them with several levels of involvement (no interaction with living animals, the presence of living animals in a terrarium, the presentation/handling of living animals by the teacher, and student interaction with living animals by touching/holding the animal in hand). The addition of spiders to the Vivarium will be one of our next projects.

Also, additional measures of trait and state and motivation can be used, such as previous experience with nature or type of pets, or examining how students with low or moderate motivation performed in the Vivarium relative to the school environment.

## 5. Conclusions

Teachers may contribute to conservation education in various ways. First, as we have shown, educational approaches in the classroom are valuable, so we suggest integrating living animals into classroom teaching [19,31]. Additionally, we suggest including practical aspects, such as handling of the animals. We should also seek out encounters with insects and arthropods, not only in school teaching but also in other ways during our lives, regardless of whether such animals are cute or not [77,78].

## Figures and Tables

**Table 1:** Invertebrate-inspired achievement test, item descriptives by testing time and treatment.

		Pre-Test						Post-Test						Follow-up-Test					
Item		Control		School		University		Control		School		University		Control		School		University	
		*M*	*(SD)*	*M*	*(SD)*	*M*	*(SD)*	*M*	*(SD)*	*M*	*(SD)*	*M*	*(SD)*	*M*	*(SD)*	*M*	*(SD)*	*M*	*(SD)*
1	Which developmental stage is located between the incomplete and the complete development of an insect?	0.02	(0.14)	0.04	(0.21)	0.19	(0.40)	0.08	(0.27)	0.13	(0.33)	0.38	(0.49)	0.20	(0.40)	0.16	(0.36)	0.41	(0.49)
2	What is mimesis?	0.05	(0.22)	0.02	(0.12)	0.07	(0.25)	0.37	(0.49)	0.58	(0.49)	0.73	(0.45)	0.21	(0.41)	0.51	(0.50)	0.66	(0.48)
3	Does the stick insect do mimesis?	0.07	(0.26)	0.08	(0.27)	0.11	(0.31)	0.40	(0.49)	0.64	(0.48)	0.79	(0.41)	0.37	(0.48)	0.68	(0.47)	0.78	(0.41)
4	A Millipede has how many legs?	0.23	(0.42)	0.29	(0.45)	0.32	(0.47)	0.53	(0.50)	0.73	(0.44)	0.79	(0.41)	0.43	(0.50)	0.71	(0.46)	0.79	(0.41)
5	A snail has how many teeth? (a) none, (b) few (<10), (c) about 10–20.	0.44	(0.50)	0.44	(0.50)	0.54	(0.50)	0.59	(0.49)	0.90	(0.30)	0.90	(0.30)	0.56	(0.50)	0.85	(0.36)	0.87	(0.33)
6	How do we call the tongue of a snail?	0.13	(0.34)	0.07	(0.25)	0.11	(0.31)	0.31	(0.46)	0.48	(0.50)	0.54	(0.50)	0.28	(0.45)	0.42	(0.49)	0.54	(0.50)
7	In which country does the hissing cockroach^3^ live?	0.05	(0.21)	0.09	(0.28)	0.11	(0.31)	0.42	(0.50)	0.61	(0.49)	0.76	(0.43)	0.26	(0.44)	0.53	(0.50)	0.68	(0.47)
8	Choose one: (a) The hissing cockroach can run fast and hiss loud. (b) The hissing cockroach cannot run and cannot hiss. (c) The hissing cockroach does only move slowly and hisses quietly. (d) The hissing cockroach can run fast, but hisses only quietly.	0.11	(0.32)	0.18	(0.39)	0.17	(0.37)	0.43	(0.50)	0.58	(0.49)	0.69	(0.46)	0.32	(0.47)	0.61	(00.49)	0.66	(0.48)
9	Which temperature range does the hissing cockroach prefer? (a) 18–23 °C, (b) 23–28 °C, (c) 28–33 °C, (d) 33–38 °C.	0.14	(0.35)	0.14	(0.35)	0.21	(0.41)	0.43	(0.50)	0.67	(0.47)	0.79	(0.41)	0.27	(0.45)	0.56	(0.50)	0.66	(0.48)

*Note:* A “don’t know” answer option was provided for each item. The items were rated 0 points (wrong answer/don’t know) or 1 point (right answer). Original items in German: 1 Welches Stadium der Insekten unterscheidet die vollständige Entwicklung der Insekten von der unvollständigen Entwicklung der Insekten? 2 Was versteht man unter Mimese? 3 Macht die Stabschrecke Mimese? 4 Wie viele Beine hat ein Tausendfüßer? 5 Wie viele Zähne hat eine Schnecke? (a) keine, (b) wenige (<10), (c) etwa 10–20. 6 Wie nennt man die Schneckenzunge? 7 Aus welchem Land stammt die Riesenfauchschabe? 8 Kreuze an: (a) Die Schabe kann schnell rennen und laut fauchen. (b) Die Schabe kann nicht rennen und nicht fauchen. (c) Die Schabe bewegt sich nur langsam und faucht leise. (d) Die Schabe kann schnell rennen, aber nur leise fauchen. 9 In welchem Temperaturbereich fühlt sich die Riesenfauchschabe wohl? (a) 18–23 °C, (b) 23–28 °C, (c) 28–33 °C, (d) 33–38 °C; 3 The translation of “Riesenfauchschabe” [“Madagascar hissing cockroach”] into the English Language gives away the answer to Item 7. Therefore, in Items 7–9, “Madagascar” was omitted.

**Table 2:** Invertebrate-inspired achievement, scale descriptives by wave and treatment.

	Control Condition			School Condition			University Condition			Total			*ICC*		Missing Values			
															On Student Level	On Class Level	Total Missing	
	*M*	*(SD)*	*n*	*M*	*(SD)*	*n*	*M*	*(SD)*	*n*	*M*	*(SD)*	*N*	Class	School			*n*	%
Achievement (pre-test)	0.14	(0.12)	243	0.15	(0.14)	984	0.20	(0.16)	598	0.16	(0.14)	1825	0.18	0.09	8	1 (28) ^2^	36	1.9
Achievement (post-test)	0.40	(0.25)	198	0.59	(0.23)	975	0.71	(0.20)	626	0.61	(0.24)	1799	0.17	0.23	19	2 (43)	62	3.3
Achievement (follow-up)	0.32	(0.22)	230	0.56	(0.22)	884	0.67	(0.22)	562	0.56	(0.25)	1676	0.18	0.23	88	5 (97)	185	9.9
Conscientiousness	3.30	(0.93)	242	3.52	(0.98)	972	3.49	(0.98)	595	3.48	(0.97)	1809	0.02	0.02	24	1 (28)	52	2.8
Interest	4.20	(0.99)	192	4.50	(0.94)	968	4.51	(0.79)	603	4.47	(0.90)	1763	0.08	0.02	33	3 (65)	98	5.3
Competence	4.10	(0.95)	190	4.12	(0.97)	967	4.08	(0.95)	603	4.11	(0.96)	1760	0.04	<0.01	36	3 (65)	101	5.4
Choice	3.87	(0.96)	190	3.92	(1.03)	962	3.91	(1.01)	603	3.91	(1.01)	1755	0.03	0.01	41	3 (65)	106	5.7
Pressure/tension	2.68	(1.20)	188	2.59	(1.25)	965	2.40	(1.12)	602	2.54	(1.20)	1755	0.04	0.03	41	3 (65)	106	5.7
Stations completed ^1^				0.84	(0.22)	971	0.88	(0.19)	626	0.86	(0.21)	1597	0.10	0.08	21	0 (0)	21	1.1

*Note: ICC* = intraclass correlation coefficient; ^1^ the control condition is excluded from the analyses in this row, because the students in the control condition did not work on any of the stations; ^2^ missing data: the numbers in brackets are the number of students in the class(es).

**Table 3:** Correlations of study variables.

		1	2	3	4	5	6	7	8
1	Achievement (pre-test)								
2	Achievement (post-test)	*** 0.26							
3	Achievement (follow-up-test)	*** 0.25	*** 0.65						
4	Conscientiousness	−0.01	*** 0.13	*** 0.14					
5	Interest	** 0.08	*** 0.26	*** 0.18	*** 0.14				
6	Competence	** 0.08	*** 0.18	*** 0.11	*** 0.14	*** 0.63			
7	Choice	** 0.08	*** 0.17	*** 0.10	** 0.08	*** 0.49	*** 0.59		
8	Pressure/tension	** −0.07	*** −0.23	*** −0.21	−0.01	* −0.05	−0.03	0.02	
9	Stations completed ^1^	*** 0.10	*** 0.39	*** 0.25	*** 0.11	*** 0.20	*** 0.19	*** 0.17	*** −0.12

*Note:* Pearson’s correlation coefficients; * *p* < 0.05, ** *p* < 0.01, *** *p* < 0.001; ^1^ the control condition is excluded from the analyses in this row, because the students in the control condition did not work on any of the stations.

**Table 4:** Invertebrate-inspired achievement by treatment and testing time (longitudinal three-level regression).

		Model 1			Model 2		
	Within Students	β	(*S.E.*)	*p*	β	(*S.E.*)	*p*
Control condition	Testing time	0.26	(0.05)	<0.001	1.98	(0.39)	<0.001
	Testing time (squared)				−1.75	(.36)	<0.001
	Snijders & Bosker’s *R*^2^	0.12			0.23		
	*N*	671					
	Level 2 clusters (student level)	243					
	Level 3 clusters (class level)	11					
School condition	Testing time	0.58	(0.02)	<0.001	3.21	(0.14)	<0.001
	Testing time (squared)				−2.67	(0.14)	<0.001
	Snijders & Bosker’s *R*^2^	0.35			0.51		
	*N*	2843					
	Level 2 clusters (student level)	992					
	Level 3 clusters (class level)	43					
University condition	Testing time	0.66	(0.04)	<0.001	3.73	(0.20)	<0.001
	Testing time (squared)				−3.11	(0.18)	<0.001
	Snijders & Bosker’s *R*^2^	0.41			0.59		
	*N*	1786					
	Level 2 clusters (student level)	626					
	Level 3 clusters (class level)	27					
Total	Testing time	0.56	(0.02)	<0.001	3.24	(0.12)	<0.001
	Testing time (squared)				−2.72	(0.11)	<0.001
	Snijders & Bosker’s *R*^2^	0.31			0.47		
	*N*	5300					
	Level 2 clusters (student level)	1861					
	Level 3 clusters (class level)	81					

*Note:* Dependent variable: invertebrate-inspired achievement; testing time: 1 = pre-test, 2 = post-test, 3 = follow-up-test.

**Table 5:** Predictors of invertebrate-inspired achievement for post-test and follow-up-test (three-level regressions).

	Invertebrate-Inspired Achievement (Post-Test)									Invertebrate-Inspired Achievement (Follow-up-Test)								
	Model 1			Model 2			Model 3			Model 1			Model 2			Model 3		
	β	(*S.E.*)	*p*	β	(*S.E.*)	*p*	β	(*S.E.*)	*p*	β	(*S.E.*)	*p*	β	(*S.E.*)	*p*	β	(*S.E.*)	*p*
Student level																		
Achievement (pre-test)	0.13	(0.03)	<0.001	0.11	(0.02)	<0.001	0.11	(0.02)	<0.001	0.13	(0.03)	<0.001	0.12	(0.03)	<0.001	0.12	(0.03)	<0.001
Gender				0.18	(0.05)	0.001	0.18	(0.06)	0.002				0.13	(0.04)	0.002	0.14	(0.05)	0.002
Conscientiousness				0.09	(0.02)	<0.001	0.07	(0.02)	0.001				0.09	(0.03)	0.001	0.08	(0.03)	0.004
Interest				0.08	(0.04)	0.034	0.05	(0.04)	0.201				0.02	(0.03)	0.583	−0.02	(0.03)	0.616
Competence				0.04	(0.04)	0.377	0.02	(0.04)	0.620				0.03	(0.05)	0.504	0.01	(0.05)	0.780
Choice				0.05	(0.03)	0.040	0.06	(0.03)	0.023				0.02	(0.03)	0.500	0.04	(0.03)	0.183
Pressure/tension				−0.11	(0.03)	<0.001	−0.09	(0.02)	<0.001				−0.11	(0.03)	<0.001	−0.10	(0.03)	0.001
Station completed							0.21	(0.03)	<0.001							0.13	(0.02)	<0.001
Class level																		
University condition	0.34	(0.14)	0.014	0.33	(0.09)	<0.001	0.27	(0.12)	0.027	0.32	(0.17)	0.056	0.27	(0.32)	0.402	0.25	(0.17)	0.153
Control condition	−0.78	(0.26)	0.003	−0.73	(0.20)	<0.001				−0.93	(0.24)	<0.001	−0.84	(0.25)	<0.001			
School level																		
School type				0.38	(0.07)	<0.001	0.32	(0.12)	0.010				0.31	(0.18)	0.081	0.33	(0.11)	0.002
*R*^2^ (student level)	0.03			0.11			0.19			0.03			0.08			0.11		
*R*^2^ (class level)	0.39			0.41			0.18			0.48			0.43			0.11		
*R*^2^ (school level)				0.93			0.58						0.89			0.94		
Snijders & Bosker’s *R*^2^	0.19			0.32			0.31			0.21			0.29			0.20		
*N*	1766			1698			1506			1642			1533			1353		
Level 2 clusters (class level)	78			77			68			75			72			63		
Level 3 clusters (school level)	34			34			30			31			31			27		

Note: Reference group: school condition; gender: 0 = male, 1 = female; school type: 0 = intermediate track, 1 = upper track; Model 3 excludes the data from the control condition.
